# Adult-Onset IgA Vasculitis Associated With Pulmonary-Renal Syndrome Following COVID-19 Infection: A Case Report and Literature Review

**DOI:** 10.7759/cureus.35527

**Published:** 2023-02-27

**Authors:** Hassan Alwafi, Deema Ashoor, Mohammad Dairi, Ghadeer Mokhtar, Khaled Dairi

**Affiliations:** 1 Department of Pharmacology and Therapeutics, Umm Al-Qura University, Makkah, SAU; 2 Department of Medicine and Surgery, Umm Al-Qura University, Makkah, SAU; 3 Department of Internal Medicine, Umm Al-Qura University, Makkah, SAU; 4 Department of Pathology and Laboratory Medicine, King Faisal Specialist Hospital and Research Centre, Jeddah, SAU; 5 Department of Internal Medicine, King Faisal Hospital, Makkah, SAU

**Keywords:** adult iga vasculitis, henoch-schönlein purpura (iga vasculitis), pulmonary-renal syndrome, sars-cov-2, covid-19

## Abstract

Immunoglobulin A (IgA) vasculitis, also known as Henoch-Schönlein purpura (HSP), is an immune complex-mediated inflammation of small blood vessels that leads to tissue destruction with or without organ damage. We described a case of a 41-year-old otherwise healthy female who presented with an ascending rash distributed on both lower extremities and arthralgia. Blood testing revealed high blood urea nitrogen (BUN), creatinine, and inflammatory markers, as well as a negative autoimmune panel. Urinalysis revealed proteinuria and hematuria. A kidney biopsy was performed, which revealed abnormalities. She was started on intravenous (IV) methylprednisolone pulse therapy. Suddenly, she complained of epistaxis and became desaturated. Computed tomography revealed bilateral pleural effusion, and she was transferred to the ICU. Bronchoalveolar lavage was performed and was consistent with an increasing bloodier return. Plasma exchange was performed. The rash and clinical symptoms improved dramatically. This study reports a case of IgA vasculitis based on The European Alliance of Associations for Rheumatology/Pediatric Rheumatology International Trials Organization/Pediatric Rheumatology European Society (EULAR/PRINTO/PRES) criteria associated with pulmonary-renal syndrome following a case of severe acute respiratory syndrome coronavirus 2 (SARS-CoV-2) infection.

## Introduction

Immunoglobulin A (IgA) vasculitis, also known as Henoch-Schönlein purpura (HSP), is an immune complex-mediated inflammation of small blood vessels, leading to tissue destruction with or without organ damage [1]. The disease affects both adults and children; however, it is rarely reported among adults [2]. It is the most prevalent kind of systemic vasculitis in children, occurring at a rate of three to 26 cases per 100,000 children each year while adults have a lower incidence of the condition, with an annual incidence of 0.1 to 1.8 per 100,000 individuals [2,3]. Males are 1.5 times more likely to get the disease than females [3].

The etiology of IgA vasculitis is unknown; however, it is thought that the condition usually occurs after a recent infection or medication use, leading to a deposition of IgA-antibody immune complexes in the small vessels (typically capillaries) of the skin, joints, kidneys, and gastrointestinal tract. Consequently, this leads to a hyper-inflammatory response [4]. The disease's presentation and progression are primarily determined by the organ involved; however, IgA vasculitis is frequently a self-limiting illness with a favorable prognosis in those who do not have renal involvement [4].

Since the emergence of COVID-19 in Wuhan, China, in 2020, the disease has been linked to several health, economic, and social complications [5]. Major health concerns have been raised over the post-coronavirus disease 2019 (COVID-19) infection, including pulmonary and extrapulmonary complications that are, in many cases, related to the hyperinflammation and cytokine storm of the disease [6]. A previous systematic review presented the clinical and laboratory factors associated with coronavirus disease based on its severity [7]. The best marker for severeness was decreased oxygen saturation (SpO2) [7]. Regarding symptoms, dyspnea, chest pain, and anorexia were relatively ominous for the seriousness of the disease and mortality [7]. Also, the study showed that critical and non-survivor groups tend to have an increase in creatine kinase (CK), C-reactive protein (CRP), procalcitonin, lactate dehydrogenase (LDH), urea, creatinine, D-dimer, prothrombin time (PT), erythrocyte sedimentation rate (ESR), interleukin-6 (IL-6), neutrophils, and total white blood cells (WBCs) and a decrease in arterial oxygen partial pressure/fractional inspired oxygen (pO2/FiO2), pO2, lymphocyte, and platelet (PLT) values when compared to other groups [7].

COVID-19-induced IgA vasculitis has been described in prior case reports [8-26]. However, these case reports were mainly in pediatrics or the very old age group [8-26]. In this report, we describe a case of a healthy adult Saudi female patient who was diagnosed with IgA vasculitis manifesting with pulmonary-renal syndrome following a recent infection with COVID-19. To our knowledge, this is the first case report of COVID-19-induced IgA vasculitis associated with pulmonary-renal syndrome among the adult age group in Saudi Arabia.

## Case presentation

A 41-year-old, medically free, married Saudi female came to the emergency department complaining of a skin rash that started in the lower extremities three days before presenting to the hospital with an ascending progression associated with bilateral knee and ankle pain. She had a history of COVID-19 infection two weeks prior to her presentation, with upper respiratory tract symptoms and a polymerase chain reaction (PCR)-confirmed diagnosis, which was treated supportively without the need for anti-viral therapy or dexamethasone. On examination, the patient was normal despite a palpable, non-blanching, pruritic rash distributed on both lower extremities. The patient was admitted for close observation.

On admission, investigations revealed a normal blood count, an elevated creatinine ratio (CRE2), and blood urea nitrogen (BUN) values of 680.8 umol/L and 39.6 mmol/L, respectively. They also revealed elevated alanine transaminase (ALT) and aspartate aminotransferase (AST) of 72 U/L and 80 U/L, respectively, a decreased serum albumin of 25 g/L, elevated inflammatory markers, an erythrocyte sedimentation rate (ESR) of 36 mm/hour, and a C-reactive protein (CRP) of 12 mg/L. Anti-nuclear antibodies (ANA), antineutrophil cytoplasmic autoantibodies (ANCA), anti-double-stranded DNA (anti-dsDNA), lupus anticoagulant, anti-glomerular basement membrane antibody (anti-GBM), hepatitis B and hepatitis C serologies, and human immunodeficiency viruses (HIV) were all negative. Immunoglobulin levels were within normal ranges, except the IgA level was elevated at 487 mg/dL. The complement level decreased, as C3 was 20.6 mg/dL and C4 was 5.43 mg/dL. Urinalysis demonstrated the presence of hematuria and massive proteinuria. Ultrasound (U/S) of the abdomen showed normal-sized kidneys with a preserved corticomedullary junction.

**Figure 1:**
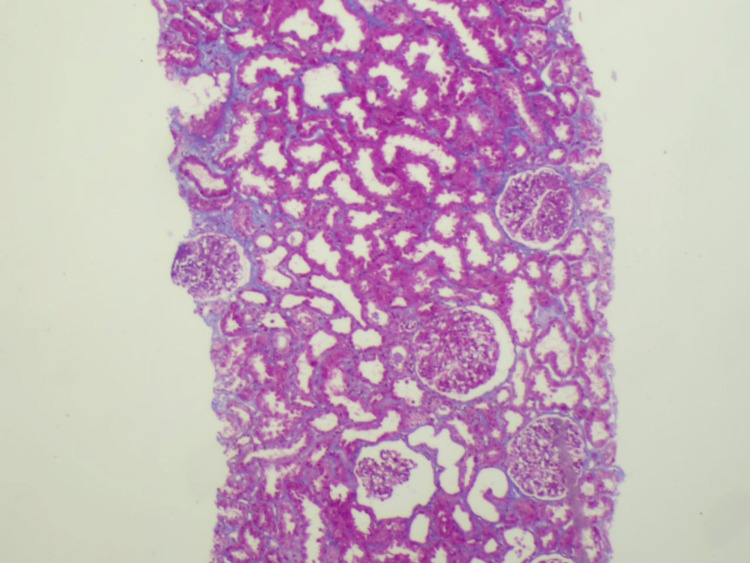
Kidney biopsy (20X, Masson trichrome stain) The tubules show mild acute tubular injury with intratubular casts. No tubular atrophy, interstitial fibrosis, or inflammation is seen

**Figure 2:**
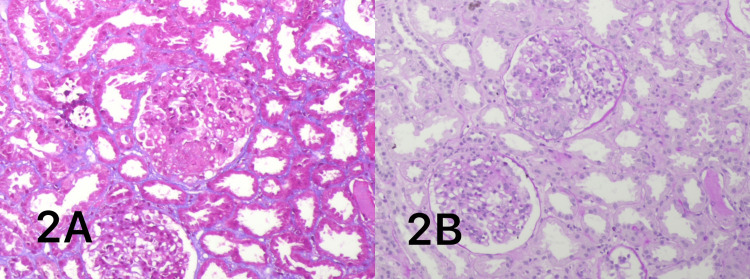
Kidney biopsy (20X, 2A: Masson trichrome stain, and 2B: periodic acid Schiff) Images of one glomerulus showing segmental karyorrhexis, fibrinoid necrosis, and cellular crescents formation

A kidney biopsy was performed, and it revealed a sufficient number of glomeruli (13), with no signs of segmental or global sclerosis. There was no glomerular hypercellularity or visible leukocyte infiltrate, but two glomeruli displayed minor segmental fibrinoid necrosis with karyorrhexis and the production of cellular crescents. Mild acute tubular damage without atrophy was visible in the tubules. There was edema in the interstitium but no inflammation or fibrosis. The blood vessels were unremarkable (Figures 1-2). The patient met the Henoch-Scholen purpura criteria and the European Alliance of Associations for Rheumatology/Pediatric Rheumatology International Trials Organization/Pediatric Rheumatology European Society (EULAR/PRINTO/PRES) criteria.

The patient was started on intravenous (IV) steroid pulse therapy methylprednisolone 1000 mg daily for three days and then switched to oral 60 mg daily. Oral hydroxychloroquine 200 mg twice daily for three days and oral calcium carbonate 600 mg twice daily for seven days were given.

**Figure 3:**
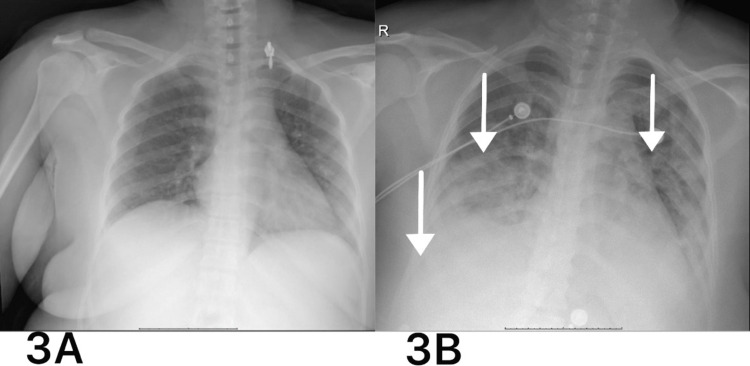
Chest X-ray 3A: Initial chest X-ray was grossly unremarkable. 3B: Repeat imaging was abnormal with evidence of bilateral hazy appearance involving the mid and lower lungs and blunting of the right costophrenic angle.

**Figure 4:**
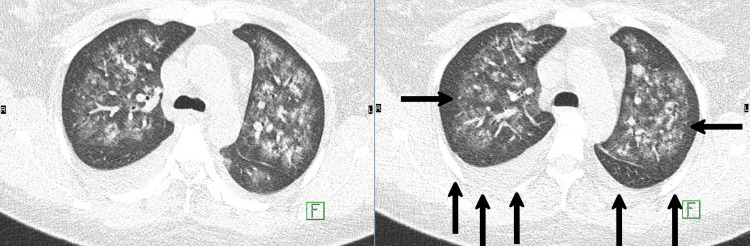
Computed tomography (CT) scan of the chest Axial CT chest at the level of the carina demonstrated extensive ground glass opacities bilaterally with subpleural sparing and small-sized pleural effusion bilaterally right greater than the left.

A few days later, she complained of epistaxis and hemoptysis. Upon examination, she had a blood pressure of 125/65 mmHg, a temperature of 37.2 °C, a regular heart rate of 105 bpm, oxygen saturation of 85% on room air, and responded to 5 l/min of supplemental oxygen via a nasal cannula, reaching 92-93%. Investigations reveal a low hemoglobin level (8.3 g/L) compared to her baseline of 12.3 g/L, low red blood cell count (3.01 x 10^12/L), low hematocrit (25.5%), and normal platelet count (320 x 10^9/L). The coagulation profile was normal, with a decreased fibrinogen level of 1.7 g/L and an elevated D-dimer level of 4.61 mg/L. An X-ray of the chest revealed bilateral pleural effusion (Figure 3). A computed tomography (CT) scan of the chest was relevant for bilateral diffuse ground-glass opacities mainly in the central area, with scattered areas of consolidation at the lower basal segments bilaterally, with bilateral pleural effusion greater on the right side (Figure 4). The patient was transferred to the intensive care unit (ICU), and bronchoalveolar lavage was performed, which was consistent with an increasing bloody return in keeping with active diffuse alveolar hemorrhage. Echocardiography revealed normal bi-ventricular systolic function, normal left ventricular diastolic function, and no hemodynamically significant valvular lesions.

The patient was started empirically on intravenous (IV) piperacillin-tazobactam while awaiting the final cultures. As discussed with the nephrologist, the patient was given two doses of intravenous immunoglobulin (IVIG), and plasma exchange was performed. The rash and clinical symptoms improved dramatically. Creatinine was reduced to 117.21 umol/L, blood urea nitrogen was normalized at 4.8 mmol/L, and hemoglobin was increased to 10.7 g/dL. The patient was discharged on a tapering schedule of oral prednisolone, and follow-up appointments have been scheduled.

## Discussion

In this case report, we describe a case of a 41-year-old female, otherwise healthy, who had a typical presentation of IgA vasculitis based on EULAR/PRINTO/PRES criteria in the context of a recent COVID-19 infection, which is thought to be the provoking factor.

The pathophysiology of IgA vasculitis is not well-understood. However, several theories arise from the fact that the disease originally occurred following environmental exposure to an infection or a medication [27]. COVID-19 is a highly contagious viral infection caused by the coronavirus 2 (SARS-CoV-2) [28]. Although SARS-CoV-2 primarily affects the respiratory system, it may also impact the gastrointestinal tract (GI) and hepatobiliary, cardiovascular, renal, and nervous systems [29]. The key mechanism is the virus's high affinity for angiotensin-converting enzyme 2 (ACE2) receptors, which are extensively expressed in most human cells and induce the direct pathway of endothelial cell damage [30,31]. The indirect pathway is produced by dysregulation of the immune response and the elevation of several inflammatory mediators and pro-inflammatory cytokines [30,31]. SARS-CoV-2 promotes an immune reaction mediated by IgA, resulting in the deposition of this IgA immune complex in multiple organs and vessels, resulting in tissue destruction with or without organ damage [5].

Patients with IgA vasculitis are treated differently based on the severity of their illness and the presence of systemic involvement [3]. Rest and analgesics are used to treat non-necrotic, non-severe purpura or arthralgias [3]. Angiotensin-converting enzyme (ACE) inhibitors are used in mild-to-moderate proteinuria and hypertension cases. In cases with systemic involvement or life-threatening manifestations, glucocorticoids alone or combined therapy with immunosuppressive drugs are used [3].

Twenty-one cases of IgA vasculitis were reported following the COVID-19 infection, as summarized in Table 1. Fourteen total pediatric cases and seven total adult cases. Two cases from the pediatric age group were reported with isolated skin manifestations that were successfully treated conservatively. Eight cases were reported with only skin and gastrointestinal tract involvement; all cases were from the pediatric age group except one; all cases treated with steroid monotherapy except one from the pediatric age group were treated conservatively with excellent outcomes. Eight cases were reported with kidney involvement but no lung involvement; all pediatric and three adult cases were treated successfully with steroid therapy. Two of the adults were given a combination of steroids and immunosuppressive therapy. Three cases were reported with combined lung and kidney involvement: two of them from the pediatric age group died from respiratory failure, and one adult was treated successfully with steroid monotherapy. Not all cases were diagnosed by biopsy, but their clinical and laboratory results met established diagnostic criteria. 

**Table 1:** Reported cases with IgA vasculitis induced by COVID-19 infection IgA: immunoglobulin A

Study	Age/Gender	Country	Covid-19 infection onset	Organs involved	Biopsy	Criteria	Treatment	Outcome
Mousavi & Jafari., 2020 [8]	6-year-old/Male	Iran	Negative at admission/clinically diagnosed	Skin, kidney, lungs, GI tract	Not performed	Not mentioned	Methylprednisolone Hydroxychloroquine.	The patient died of respiratory failure
Allez et al., 2020 [9]	24-year-old/Male	France	Positive at admission	Skin and GI tract	Skin Biopsy: IgA vasculitis, with perivascular and vessel wall infiltration by neutrophils and lymphocytes. DIF: leukocytoclasia, and C3 and IgA deposits in dermal capillaries	Not mentioned	Methylprednisolone	Not mentioned
Gurzu et al., 2020 [10]	13-month-old/Male	Romania	Clinically diagnosed/ not confirmed with PCR	Kidneys and lungs	Autopsy: Lungs: bilateral diffuse alveolar damage with severe desquamation of type II alveolocytes and intra-alveolar macrophages. The bronchial epithelium was focally damaged with positive IgA deposition Kidneys: enlargement of the mesangium with positive IgA deposition	Not mentioned	Supportive care	Death due to the sudden respiratory compromise (desquamative interstitial pneumonia)
Suso et al., 2020 [11]	78-year-old/Male	Spain	Negative at admission/ symptoms start to appear three weeks after Covid-19 infection	Skin and Kidney	Kidney biopsy: 7 glomeruli were identified to be pathologic DIF: IgA granular deposits in the glomerular mesangium Skin Biopsy: cutaneous vasculitis	Not mentioned	Treatment: Methylprednisolone Rituximab Prednisone	Improvement of the rash and serum creatinine but persisted proteinuria and hematuria
Huang et al., 2020 [12]	65-year-old/Female	China	Positive at admission	Kidneys and lungs	Kidney biopsy: 16 glomeruli were identified to be diseased DIF: +2 glomeruli positive for IgA	Oxford score of M0E0S1T1C1	Methylprednisolone	Improvement of the symptoms and renal function
Nakandakari Gomez et al., 2021 [13]	4-year-old/Female	Peru	Positive at admission	GI tract and skin	Not performed	Not mentioned	Dexamethasone Prednisone	Improvement of abdominal pain and purpuric rash
Hoskins et al., 2021 [14]	2-year-old/Male	United States of America	Positive at admission	Skin and GI tract	Biopsy of gastric mucosa: gastritis Skin biopsy: Superficial perivascular inflammation with neutrophils, concerning for vasculitis DIF: positive for IgA	Not mentioned	Intravenous steroids Oral steroid	Complete resolution of skin findings and abdominal symptoms
Sandhu et al., 2021 [15]	22-year-old/Male	India	Positive at admission	Skin, joints, and Kidney	Skin biopsy: Leukocytoclastic vasculitis. DIF: negative Kidney biopsy: Focal necrotizing, mesangial, and focal endocapillary proliferative IgA nephropathy with mesangial granular deposits of IgA	Not mentioned	Dexamethasone Prednisolone Mycophenolate mofetil	Cutaneous lesions, joint involvement, and abdominal symptoms resolved
AlGhoozi & AlKhayyat., 2021 [16]	4-year-old/Male	Bahrain	Negative at admission/symptoms start to appear 37 days after Covid-19 infection	Skin and joints	Not performed	EULAR/PRINTO/PRES	Paracetamol	Joint pain resolved Rash was still present bilaterally in the lower limbs
Jacobi et al., 2021 [17]	3-year-old/Male	Israel	Positive at admission	Skin and GI tract	Not performed	Not mentioned	Methylprednisolone	Improvement of abdominal pain and purpuric rash
Li et al., 2021 [18]	30-year-old/Male	Canada	Positive at admission	Skin, joints, Kidney, and GI tract	Skin biopsy: Neutrophil-rich small-vessel vasculitis, suggestive of leukocytoclastic vasculitis. DIF: negative Kidney biopsy: Focally crescentic and segmentally necrotizing IgA with focal endocapillary hypercellularity	Not mentioned	Prednisone	Resolution of rash, abdominal, and joint pain. Improvement of urinalysis findings
Falou et al., 2021 [19]	8-year-old/Male	Lebanon	Positive at admission	Skin and joints	Not performed	EULAR/PRINTO/PRES	NSAIDs and paracetamol	complete resolution of rash and joint pain.
Barbetta et al., 2021 [20]	62-year-old/Male	Italy	Positive at admission	Skin, Kidneys, and GI tract	Skin biopsy: Perivascular and interstitial lymphocytic infiltrate mainly distributed in the upper dermis, together with extravasated red blood cells, ectasia capillary vessels, and endothelial cells with signs of swelling without atypia. DIF: intense IgA vascular deposits	Not mentioned	Methylprednisolone	Improvement of renal function, abdominal pain, and skin rash.
Kumar et al., 2021 [21]	13-year-old/Male	United States of America	Negative at admission/ symptoms start to appear 4 weeks after Covid-19 infection	Skin and kidneys	Skin biopsy: Small vessel neutrophilic vasculitis. Non-specific, patchy deposition of fibrinogens present in the superficial dermis only. DIF: negative	Not mentioned	Prednisolone	Gradually improved
el Hasbani et al., 2021 [22]	16-year-old/Male	Lebanon	Positive at admission	Skin, kidneys, and GI tract	Not performed	EULAR/PRINTO/PRES	Prednisolone	Improvement of the rash and urinalysis
Borocco et al., 2021 [23]	13-year-old/Female	France	Positive at admission	Skin and GI tract	Not performed	EULAR/PRINTO/PRES	Pain relievers	No complications
Jedlowski & Jedlowski., 2022 [24]	70-year-old/Male	United States of America	Positive at admission	Skin, kidneys, and GI tract	Skin biopsy: Leukocytoclastic vasculitis DIF: strong signal granular IgA deposition Kidney biopsy: Mesangial hypercellularity, focal/mild endocapillary hypercellularity, tubular atrophy, interstitial fibrosis, and lymphocytic tubulitis, without crescents. DIF: granular mesangial deposition of IgA (2+), with identification of patchy effacement of podocytes	Not mentioned	Methylprednisolone Prednisone	Resolution of abdominal pain, rash, and renal function.
Ziyara et al., 2022 [25]	12-year-old/Male	United Kingdom	Positive at admission	Skin, joints, and GI tract	Not performed	Royal College of Pediatrics and Child Health Guidelines 2020	Prednisolone	Improvement of skin rashes Developed self-limited abdominal pain with painful swellings of the small joints in the hands bilaterally with normal investigations
Asiriet al., 2022 [26]	4-year-old/Male	Saudi Arabia	Positive at admission	Skin, joints, and kidney	Not performed	Not mentioned	Prednisolone	Improvement of skin rashes and joint pain
	23-month-old/Male		Positive at admission	Skin, joints, and GI tract	Not performed	Not mentioned	Prednisolone	Improvement of the GI symptoms, joint pain, and skin rash
	4-year-old/Male		Positive at admission	Skin, joints, and GI tract	Not performed	Not mentioned	Prednisolone	Improvement of the GI symptoms, joint pain

Our patient was diagnosed with IgA vasculitis based on the EULAR/PRINTO/PRES criteria associated with pulmonary-renal syndrome following a COVID-19 infection. Initially, the patient was treated with steroids to reduce the risk of developing persistent kidney disease because urine protein levels were significantly elevated. However, these alone did not provide the effectiveness desired, and she was later successfully treated with plasma exchange and intravenous immunoglobulin (IVIG).

As COVID-19 is a novel disease and its pathogenic mechanism of causing IgA vasculitis is not well understood, every patient who is infected with or recently recovered from COVID-19 and presents with a skin rash or arthralgia should have baseline blood and urine tests done and should be treated promptly to avoid the emergence of irreversible consequences.

It's vital to remember that case reports are descriptive and can't be utilized to prove a cause-and-effect link. On the other hand, the efficacy of current therapeutic regimens is debatable since the majority of available research has been conducted on children, with findings that have been generalized to adults. As a result, we recommend further studies be done to be able to determine the role of SARS-CoV-2 in the pathogenesis of IgA vasculitis and the efficacy of the available therapeutic interventions on adults.

## Conclusions

In conclusion, our study showed that COVID-19 could serve as an indirect trigger of IgA vasculitis through the hyperinflammatory response and cytokine storm. Adult-onset IgA vasculitis may be efficiently treated with plasma exchange and intravenous immunoglobulin. We present the first case of IgA vasculitis based on the EULAR/PRINTO/PRES criteria associated with pulmonary-renal syndrome after a SARS-CoV-2 infection that was effectively treated with intravenous immunoglobulin (IVIG) and plasma exchange and showed an impressive response.

## References

[1] Baigrie D, Goyal A, Crane JS (2022). Leukocytoclastic Vasculitis. https://pubmed.ncbi.nlm.nih.gov/29489227/..

[2] Piram M, Mahr A (2013). Epidemiology of immunoglobulin A vasculitis (Henoch-Schönlein): current state of knowledge. Curr Opin Rheumatol.

[3] Maritati F, Canzian A, Fenaroli P, Vaglio A (2020). Adult-onset IgA vasculitis (Henoch-Schönlein): Update on therapy. Presse Med.

[4] Roache-Robinson P, Hotwagner DT (2022). Henoch Schönlein Purpura. https://pubmed.ncbi.nlm.nih.gov/30725937/.

[5] Wong K, Farooq Alam Shah MU, Khurshid M, Ullah I, Tahir MJ, Yousaf Z (2022). COVID-19 associated vasculitis: a systematic review of case reports and case series. Ann Med Surg (Lond).

[6] Tan LY, Komarasamy TV, Rmt Balasubramaniam V (2021). Hyperinflammatory immune response and COVID-19: a double edged sword. Front Immunol.

[7] Minh LH, Abozaid AA, Ha NX (2021). Clinical and laboratory factors associated with coronavirus disease 2019 (Covid-19): a systematic review and meta-analysis. Rev Med Virol.

[8] Mousavi MS, Jafari M (2020). COVID-19 in IgA vasculitis. Iran J Pediatr.

[9] Allez M, Denis B, Bouaziz JD (2020). COVID-19-related IgA vasculitis. Arthritis Rheumatol.

[10] Gurzu S, Satala CB, Melit LE (2020). COVID-19 like findings in a fatal case of idiopathic desquamative interstitial pneumonia associated with IgA glomerulonephritis in a 13-month-old child. Front Pediatr.

[11] Suso AS, Mon C, Oñate Alonso I (2020). IgA vasculitis with nephritis (Henoch-Schönlein Purpura) in a COVID-19 patient. Kidney Int Rep.

[12] Huang Y, Li XJ, Li YQ (2020). Clinical and pathological findings of SARS-CoV-2 infection and concurrent IgA nephropathy: a case report. BMC Nephrol.

[13] Nakandakari Gomez M, Marín Macedo H, Seminario Vilca R (2021). IgA (Henoch Schönlein Purpura) vasculitis in a pediatric patient with COVID-19 and strongyloidiasis. Rev Fac Med Hum.

[14] Hoskins B, Keeven N, Dang M, Keller E, Nagpal R (2021). A child with COVID-19 and immunoglobulin A vasculitis. Pediatr Ann.

[15] Sandhu S, Chand S, Bhatnagar A, Dabas R, Bhat S, Kumar H, Dixit PK (2021). Possible association between IgA vasculitis and COVID-19. Dermatol Ther.

[16] AlGhoozi DA, AlKhayyat HM (2021). A child with Henoch-Schonlein purpura secondary to a COVID-19 infection. BMJ Case Rep.

[17] Jacobi M, Lancrei HM, Brosh-Nissimov T, Yeshayahu Y (2021). Purpurona: a novel report of COVID-19-related Henoch-Schonlein purpura in a child. Pediatr Infect Dis J.

[18] Li NL, Papini AB, Shao T, Girard L (2021). Immunoglobulin-A vasculitis with renal involvement in a patient with COVID-19: a case report and review of acute kidney injury related to SARS-CoV-2. Can J Kidney Health Dis.

[19] Falou S, Kahil G, Merhi BA, Dana R, Chokr I (2021). Henoch Schonlein purpura as possible sole manifestation of COVID-19 in children. Acta Sci Paediatr.

[20] Barbetta L, Filocamo G, Passoni E, Boggio F, Folli C, Monzani V (2021). Henoch-Schönlein purpura with renal and gastrointestinal involvement in course of COVID-19: a case report. Clin Exp Rheumatol.

[21] Kumar G, Pillai S, Norwick P, Bukulmez H (2021). Leucocytoclastic vasculitis secondary to COVID-19 infection in a young child. BMJ Case Rep.

[22] El Hasbani G, Taher AT, Jawad AS, Uthman I (2021). Henoch-Schönlein purpura: another COVID-19 complication. Pediatr Dermatol.

[23] Borocco C, Lafay C, Plantard I, Gottlieb J, Koné-Paut I, Galeotti C (2021). SARS-CoV-2-associated Henoch-Schönlein purpura in a 13-year-old girl. Arch Pediatr.

[24] Jedlowski PM, Jedlowski MF (2022). Coronavirus disease 2019-associated immunoglobulin A vasculitis/Henoch-Schönlein purpura: a case report and review. J Dermatol.

[25] Ziyara R, Thompson A, Liu B (2022). Henoch-Schönlein purpura in a COVID-19-positive child with abdominal pain and PIMS-TS. Clin Pediatr (Phila).

[26] Asiri A, Alzahrani F, Alshehri S, Hassan AbdelQadir Y (2022). New-onset Henoch-Schonlein purpura after COVID-19 infection: a case report and review of the literature. Case Rep Pediatr.

[27] Sugino H, Sawada Y, Nakamura M (2021). IgA vasculitis: etiology, treatment, biomarkers and epigenetic changes. Int J Mol Sci.

[28] Cascella M, Rajnik M, Aleem A, Dulebohn SC, di Napoli R (2022). Features, Evaluation, and Treatment of Coronavirus (COVID-19). http://pubmed.ncbi.nlm.nih.gov.

[29] Gupta A, Madhavan MV, Sehgal K (2020). Extrapulmonary manifestations of COVID-19. Nat Med.

[30] Gavriatopoulou M, Korompoki E, Fotiou D (2020). Organ-specific manifestations of COVID-19 infection. Clin Exp Med.

[31] Liu Y, Sawalha AH, Lu Q (2021). COVID-19 and autoimmune diseases. Curr Opin Rheumatol.

